# Unraveling the Complexity of Asthma: Insights from Omics Approaches

**DOI:** 10.3390/biomedicines12051062

**Published:** 2024-05-11

**Authors:** Esther Herrera-Luis, Natalia Hernandez-Pacheco

**Affiliations:** 1 Department of Epidemiology, Bloomberg School of Public Health, Johns Hopkins University, Baltimore, MD 21205, USA; eherre11@jh.edu; 2 Department of Clinical Science and Education, Södersjukhuset, Karolinska Institutet, 11883 Stockholm, Sweden; 3 CIBER de Enfermedades Respiratorias (CIBERES), Instituto de Salud Carlos III, 28029 Madrid, Spain

Asthma is a heterogeneous respiratory disease that represents a substantial social and economic burden [1] and is highly detrimental to the quality of life of both patients and their caregivers [2]. It is the most common chronic disease in children and adolescents [2]. Early therapeutic interventions are critical, as severe symptoms are common at these stages of the life course [3]. Additionally, severe childhood asthma accompanied by a substantial decline in lung function is an important risk factor for the persistence of asthma and the development of other chronic airway diseases during adulthood [4,5,6].

Asthma is a complex condition resulting from numerous interactions between endogenous and exogenous factors [7,8]. To date, most research efforts have focused on unraveling the genetic determinants and environmental factors that play a key role in asthma [9]. Nonetheless, our understanding of the mechanisms underlying this condition and related traits remains incomplete [10], most likely because asthma encompasses a diverse range of pathophysiological mechanisms and variable clinical manifestations [2]. As a result, despite the widespread application of genomics in asthma research [11], we continue to lag far behind in translating its findings into the clinical setting [12]. Moving towards a treatable traits perspective in chronic airway disease could further address the complexity of asthma, as well as patient heterogeneity [13]. Novel opportunities for genetic risk prediction in asthma have also arisen as a result of the recent development of multi-ancestry polygenic risk scores (PRSs) that aggregate the association effect of numerous variants across the genome [14,15]. Nonetheless, other omics remain underrepresented in this field; this is especially true with regard to the concept of combining several data layers through a multiomic strategy [16,17]. These may substantially contribute to the accurate diagnosis of asthma and the development of novel, effective preventive and management strategies, which can be personalized to each patient. Hence, this Special Issue focuses on recent advances in the application of omics approaches to investigate asthma and related traits.

The first work by Martin-Almeida et al. (2023) explores the blood DNA methylome of bronchodilator drug response (BDR) and fractional exhaled nitric oxide (FeNO) in a multi-ethnic pediatric cohort with moderate-to-severe asthma [18]. The authors identify several significant differentially methylated regions (DMRs) enriched in genes involved in aging and immune pathways. Moreover, the CpG cg12835256 (*PLA2G12A*) reaches genome-wide significance for FeNO and is replicated in nasal epithelial cells. *PLA2G12A* encodes for the phospholipase A2 Group XIIA, which is implicated in bronchial hyperresponsiveness and eicosanoid synthesis in asthma [19]. These data argue in favor of prior evidence of the potential role of eicosanoids as mediators of airway inflammation in asthma [20].

Secondly, Logoń et al. [21] provide an elegant state-of-the-art summary of the investigation of the microbiome in asthma pathogenesis and treatment. The authors thoroughly describe the importance of the gut and airway microbiota composition in the development and modulation of the immune response, as well as asthma susceptibility and treatment response. Furthermore, metabolites produced by commensal gut bacteria have antibiotic effects against pathogenic microorganisms. Therefore, an altered composition and functionality of the intestinal microbiota caused by environmental exposures across the life course, but most importantly, during infancy and childhood, might be involved in an imbalance in bacterial species and the alteration of the inflammatory response and asthma risk [21]. On the other hand, the bronchial microbiota composition has shown a strong association with a differential response to conventional asthma treatments [22,23]. For instance, enrichment in *Haemophilus* and bacteria of the *Actinobacteria* phylum has been associated with an increased response to inhaled corticosteroids (ICS), whereas Proteobacteria have been linked to poorer asthma control [22,23]. Additionally, prebiotic use, probiotics, dietary interventions, and fecal microbiota transplantation have been proposed as promising prophylactic or therapeutic strategies in asthma, though these approaches require further investigation [21].

Thirdly, Shailesh et al. explore (T2) mechanisms in asthma, as well as lung mechanics and function, by mining evidence from human studies and animal models described in the literature [24]. The authors emphasize the intricate interaction between obesity and asthma, highlighting the critical necessity of elucidating the relevant mechanisms, which are currently poorly understood. In particular, the authors describe the role of insulin resistance in triggering airway hyper-responsiveness in asthma. In this context, diabetes medications such as metformin and glucagon-like peptide 1 receptor (GLP-1R) agonists are promising treatments for obesity-associated asthma. Nonetheless, further research into the molecular mechanisms underlying the impact of obesity on asthma severity and randomized clinical trials to assess the efficacy of these drugs are warranted. On the other hand, obesity-related gut microbial dysbiosis and microbiome-derived short-chain fatty acids may influence immune dysregulation in asthma. Indeed, both human studies and animal models have demonstrated that a fiber-rich diet may significantly reduce asthma pathogenesis in obese asthmatic individuals through microbiome regulation. Moreover, the authors highlight the urgent need to investigate the molecular mechanisms underlying obesity-associated non-T2 factors in asthma to prioritize drug targets for potential treatment of obesity-associated asthma [24].

In summary, this Special Issue describes recent advances in asthma research through the application of promising omics approaches, evidencing their high potential for shortening the path toward obtaining a complete picture of the underlying molecular mechanisms of diverse asthma-related traits and the development of accurate diagnostic and management strategies.
